# Robotic-assisted gastrectomy for gastric cancer: a European perspective

**DOI:** 10.1007/s10120-019-00979-z

**Published:** 2019-07-04

**Authors:** Gijsbert I. van Boxel, Jelle P. Ruurda, Richard van Hillegersberg

**Affiliations:** Department of Surgery, G04 228, University Medical Centre Utrecht, Heidelberglaan 100, 3584 CX Utrecht, The Netherlands

**Keywords:** Gastric cancer, Robotic-assisted gastrectomy, RAG, Outcomes

## Abstract

Gastrectomy is the mainstay treatment for gastric cancer. To reduce the associated patient burden, minimally invasive gastrectomy was introduced in almost 30 years ago. The increase in the availability of surgical robotic systems led to the first robotic-assisted gastrectomy to be performed in 2002 in Japan. Robotic gastrectomy however, particularly in Europe, has not yet gained significant traction. Most reports to date are from Asia, predominantly containing observational studies. These cohorts are commonly different in the tumour stage, location (particularly with regards to gastroesophageal junctional tumours) and patient BMI compared to those encountered in Europe. To date, no randomised clinical trials have been performed comparing robotic gastrectomy to either laparoscopic or open equivalent. Cohort studies show that robotic gastrectomy is equal oncological outcomes in terms of survival and lymph node yield. Operative times in the robotic group are consistently longer compared to laparoscopic or open gastrectomy, although evidence is emerging that resectional surgical time is equal. The only reproducibly significant difference in favour of robot-assisted gastrectomy is a reduction in intra-operative blood loss and some studies show a reduction in the risk of pancreatic fistula formation.

## Introduction

Gastric cancer is the 5th most common cancer globally with a crude incidence of 13.5/100.000 in the population. Incidence varies widely between continents and the disease is far more prevalent in the Far East. To illustrate this, the crude incidence of gastric cancer is 10/100000 in The Netherlands compared to 90/100000 in Japan. Globally, gastric cancer is the third most deadly cancer annually [1]. The mainstay of curative treatment is surgical resection and lymphadenectomy with or without (neo)adjuvant therapy based on the stage of the disease and patient co-morbidity.

Minimally invasive surgery is associated across many surgical specialties with a reduction in post-operative pain, hospital length of stay and faster return to normal activities of daily living. Laparoscopic-assisted gastrectomy (LAG) has an established role in early, and increasingly locally advanced, gastric cancers since it was first described in 1994 [2]. The Japanese gastric cancer treatment guideline now recommends distal LAG for early gastric cancers in part based on the KLASS-01 study [3] and multiple meta-analyses [4–8]. The benefit for locally advanced cancers had until very recently not been proven by RCT, but Lee et al. have now published on the 3-year follow-up of the KLASS-02 trial [9]. This has shown that distal LAG with D2 lymphadenectomy for locally advanced gastric cancer has benefits in terms of lower complication rate, faster recovery, and less pain compared with open gastrectomy (OG). Cui et al. published an RCT comparing LAG and OG which included approximately one-third of total gastrectomies (42/128 LAG and 39/142 OG) amongst a majority of partial or distal resections. A subgroup analysis of this cohort focussing on OG total gastrectomy versus LAG total gastrectomy again showed similar benefits for LAG at no oncological expense [10]. A large multicentre Dutch randomised study, LOGICA, has just finished recruiting exclusively comparing total OG to total LAG [11]. Results are awaited in the second half of 2019.

Over the past 2 decades, robotic-assisted surgery has witnessed a meteorological rise in its uptake and applications [13]. The drivers for the ongoing and increasing innovation originate from the desire to offer greater operative precision that may translate into enhanced clinical outcomes. Robotic-assisted gastrectomy (RAG) for gastric cancer was first reported in 2002 [14]. A relatively recent publication from South Korea, one of the greatest developers and adopters of RAG, shows that the uptake is increasing exponential though only representing approximately 4% of the total robotic procedures performed annually [15], which represents about 2% of the gastrectomies performed in South Korea [16]. The premise is that RAG is expected to deliver at least the same benefits of laparoscopic surgery compared to open, but in addition, due to 3-dimensional vision, high magnification, increased degrees of freedom including endo-wristed instrumentation, stable optical platform and tremor reduction technology, potentially be superior to established minimally invasive methods. This could result in reducing a surgeon’s learning curve and creating improved training. To date, however, prospective multicentre randomised studies remain lacking, and evidence comprises several case-matched series [e.g. 17–19] and meta-analyses [12, 20–24]. However, the majority of the meta-analyses report on a small number of Asian cohort studies and often compare RAG to both LAG and OG. Furthermore, the difference in complexity and associated morbidity between distal gastrectomy and total gastrectomy is not always specified in comparative studies and this must be considered when interpreting the perceived lack of benefit for RAG.

This article reviews the current evidence base for robotic-assisted gastrectomy for gastric cancer. Oncological outcomes, potential benefits, complications, limitations and cost will all be discussed. The available evidence will be related to European cohorts, in whom there is relatively little known about the outcomes of RAG since only few comparative studies have been performed.

## The operation

Minimally invasive gastrectomy follows the same oncological principles as those established for open surgery. Song et al. eloquently describe the procedure (both distal and total gastrectomy) using the da Vinci 4 arm system (Intuitive Surgical Inc, Sunnyvale, CA, USA) in great detail in a review of 100 sequential cases [25]. Patients are commonly placed supine in 15 degrees anti-Trendelenburg position. Four robot ports are placed and one assistant port across the above the midline of the abdomen in both quadrants.

The case series reported in the literature to date all utilise the Intuitive Surgery da Vinci system. Robotic surgery aims to address many of the ergonomically and optical disadvantages of laparoscopy. The operative field is magnified tenfold and allows the primary (console) surgeon better optical control through the high-definition 3-D views from a mounted, stabilized surgeon-controlled camera reducing reliance on an assistant surgeon. Furthermore, robotic surgical tools allow flexible, endo-wristed movement capabilities, self-assistance and retraction through a third operating robotic arm. The improved surgical dexterity and ergonomics provided by the robot result from the instruments’ 7 degrees of freedom, 90° articulation, and 540° rotation, permitting manipulation within small spaces. Although this is particularly relevant in a confined area such as the chest, hiatal dissection and lymphadenectomy on the superior border of the pancreas are certainly aided by this. Several studies have shown that the robot can enhance dexterity by up to 65%, reduce skill-based errors by 93%, and reduce the time needed to complete a given task by 40% [26, 27].

Earlier da Vinci system was more confined in terms of the operative field attainable without further port placement and/or re-docking. Gastrectomy requires abdominal surgical access from relatively deep into the diaphragmatic hiatus, the splenic hilum, the duodenum and the retro-colic space. Essentially this requires access to 3 quadrants of the abdomen, which until recently has proven challenging robotically. The new Xi system with its slim arms and rotating boom (and anticipated systems such as the Versius, [28], and Verb [29] with either independent arms or table mounted arms) are marketed to permit greater access range and multi-quadrant use.

## Short-term surgical outcomes

### Operative time and learning curve

The operative time for RAG is longer than LAG [24] (mean 58 min) which in turn is longer then open surgery (mean 64 min) [30], therefore, making RAG approximately 2 h longer than OG. The reasons for this are multiple, but the docking of the robot can be time consuming, particularly in theatres that use the robot intermittently. Most studies that make the distinction between docking time and console time report this to be in the order of 20 min. A recent study by Liu et al. [31] specifically investigated the time taken to perform distal gastrectomy by robotic and laparoscopic means. This retrospective study included 10 cases in the RAG and LAG groups and, once again, showed the total procedure time for RAG to be on average 57 min longer than LAG. It did, however, assess specific resectional components of the operation and showed that the actual surgical time was the same in the RAG and LAG groups. The conclusion was, therefore, that robotic-assisted surgery still incurs “junk” time, which may be addressed by simpler system setup, faster instrument changes and more sophisticated energy devices. In our own experience, we found that the average docking time/operative time reduced over 25 procedures and subsequently stabilised to 12–15 min [27]. The literature disproportionally reports on early experiences of RAG as take up is still relatively low. Nonetheless, these series are often compared to established open or laparoscopic methods where surgeons are many cases beyond there learning curve. Only a few studies formally assess their learning curve, which has been reported between 20 and 95 [25, 32–34] cases, depending on previous experience in gastrectomy. As such, it can be expected that many of the current operative times reported are not reflective of the optimal operative time a given surgeon can achieve.

### Blood loss

A reduction in intra-operative blood loss has been the most consistent finding in the meta-analyses published on RAG to date. This is the case for those studies assessing RAG vs LAG and those that compare RAG with OG. The most recent study reports a mean reduction of blood loss of 23.7 ml for RAG compared to laparoscopic or open surgery [24]. Although this number is relatively low, differences in blood loss should not be disregarded as unimportant as there is certainly evidence of reduced peri-operative infection rates associated with reduced blood loss [35] and potentially even recurrence rates [36].

### Pancreatic fistula

Pancreatic damage due to manipulation during dissection and D2 lymphadenectomy may result in pancreatic leaks and fistula formation. Although a relatively rare complication of gastrectomy, the morbidity associated with this can be significant. The introduction of LAG has led some groups to suggest that the incidence of pancreatic trauma is greater [37–39]. There have even been reports of specialised equipment being used to reduce the risk of pancreatic fistula [40], but some believe robotic dissection may reduce pancreatic damage by facilitating careful, yet radical, resection around the border of the pancreas based on their case series [41–43]. Guerra et al. [44] recently published a meta-analysis investigating the incidence of pancreatic complications between RAG and LAG, particularly looking at post-operative pancreatic fistula formation and acute pancreatitis. Although there was a trend towards fewer pancreatic complications with RAG, (with an OR of 0.8), this was not statistically significant. The review included 4 new case series not previously included in meta-analyses, one of which [42] showed a significant difference between pancreatic complications between RAG (10%) and LAG (22.5%). This study was specifically designed to formally assess pancreatic complications using drain and serum amylase and is worthy of separate mention beyond the aforementioned meta-analysis. The rationale for the difference, according to the authors, was the constant careful retraction achieved robotically, which is not replicated by a human first assistant. A recent study by Uyama et al. specifically assessed the morbidity of RAG with a historic control [45]. This multicentre, prospective, single arm study showed that pancreatic leaks occurred in 5.9% of patients, although the majority required no intervention. This reflects the findings of previous retrospective comparative studies.

### Conversion to open and peri-operative mortality or anastomotic leak

There has been no significant difference in any of the studies published to date in terms of conversion to open or laparoscopy or re-operation following index surgery. Equally, peri-operative mortality (both 30 and 90 days) are not significantly different between RAG, LAG and OG (0.4–0.6% [24]). There was no difference observed in anastomotic leak rates between any of the groups.

## Oncological outcomes

### Cancer stage

The largest Far Eastern single-centre study of RAG reflects the fact that gastric cancer is detected earlier with consequently lower T stages and the majority being N0 [46]. This compares to a majority of Stage III disease (35%) in the largest available sole European cohort [47]. As such the available data, particularly in terms of resection margins, survival and conversions rates need to be carefully weight up when wanting to utilise evidence in a substantially different population.

### Lymph node yield

Lymphatic drainage of the stomach has been extensively studied since the 1950s and the Japanese were the first to classify regional gastric lymph nodes into 16 stations which were widely adopted worldwide [48]. In 1997, the Japanese Gastric Cancer Association further defined and subdivided the nodal stations into 20 stations and a further 3 in the thorax (110, 111, 112) [49]. The nodes are further divided into tiers reflecting that certain stations lie outside the operative field (e.g. station 16b2; para-aortic) which would, therefore, represent distant metastases. Three types of lymphadenectomy are described in the context of oncological gastric resections; D1, D1 + and D2. For total gastrectomy, the lymph node stations to be dissected in D1 lymphadenectomy are stations from No. 1 to 7; D1 + includes D1 stations plus station 8a, 9, and 11p, and D2 includes D1 stations plus stations No. 8a, 9, 10 (although this remains under debate), 11p, 11d, and 12a. For tumours invading the oesophagus, D1 + includes Nos. 110 and D2 includes Nos. 19, 20, 110 and 111. A great deal of research has been performed to establish the oncological benefit of the various lymphadenectomy. The 15-year follow-up of the Dutch D1-D2 trial, randomising patients to a D1 or D2 lymphadenectomy, definitively showed a survival benefit for (spleen preserving) D2 lymphadenectomy, although associated with larger peri-operative morbidity and mortality [50]. With regard to RAG, the lymph node yield has not been significantly different in any of the meta-analyses published compared to LAG or OG. The only cohort studies that have shown a difference in favour of RAG were Cianchi et al. 2016 [51] and, in the context of spleen preserving D2 total gastrectomy, an increase in splenic artery nodes [52]. One study [53] has shown a benefit in “obese’ patients in terms of improved lymph node yield, but the mean BMI in this cohort was 27 (see Tables 1, 2). Conversely, Hyun showed the opposite with a decreased lymph node yield in “obese” patients for RAG [54]. Caruso et al. [55] showed a decreased lymph node yield for RAG when comparing it to OG.

**Table 1:** Eastern studies included in the meta-analyses on RAG performed to date

Study	Number of RAG (Total in study)	Type of RAG resection	% early tumours (< T2N1)	Country	BMI (mean kg/m^2^)	Outcome summary
Song et al. (2009) [25]	40 RAG 20 LAG 20	Subtotal	100	South Korea	23	Increased operative time RAG
Kim et al. (2010) [82]	39 RAG 12 LAG 11 OG 16	Subtotal	100	South Korea	21	Increased operative time RAG Reduced blood loss and LOS RAG
Woo et al. (2011) [62]	827 RAG 236 LAG 591	Subtotal (73%) Total (27%)	93	South Korea	23	Increased operative time RAG Reduced blood loss RAG
Kim et al. (2012) [83]	5839 RAG 436 LAG 861 OG 4542	Subtotal (75%) Total (25%)	86	South Korea	24	Increased operative time RAG Makes comparisons between MIG and OG; increased anastomotic leak in MIG, reduced post op ileus and LOS in MIG
Park et al. (2012) [32]	150 RAG 30 LAG 120	Subtotal	100	South Korea	24	Increased operative time RAG Reduced performance status and drain output in RAG
Eom et al. (2012) [84]	92 RAG 30 LAG 62	Subtotal	92	South Korea	24	Increased operative time RAG, smaller proximal margin RAG and increased cost
Huang et al. (2012) [85]	689 RAG 39 LAG 64 OG 586	Subtotal (96%) Total (4%)	93	Taiwan	24	Reduced blood loss and LOS RAG Increased operative time RAG
Uyama et al. (2012) [86]	250 RAG 25 LAG 225	Subtotal	100	Japan	22	Decreased LOS RAG
Kang et al. (2012) [87]	382 RAG 100 LAG 282	Subtotal (84%) Total (16%)	93	South Korea	24	Increased LOS for RAG Reduced blood loss RAG
Yoon et al. (2012) [88]	101 RAG 36 LAG 65	Total	100	South Korea	23	Increased operative time RAG
Hyun et al. (2013) [54]	121 RAG 38 LAG 83	Subtotal (76%) Total (24%)	87	South Korea	24	RAG group statistically younger Subgroup analysis of “obese” pts (BMI > 25) showed reduced lymph node yield in RAG
Son et al.(2014) [52]	109 RAG 51 LAG 58	Total	74	South Korea	23	Increased operative time RAG Increased lymph node yield along splenic artery RAG
Noshiro et al. (2014) [43]	181 RAG 21 LAG 160	Subtotal	85	Japan	23	Reduced LOS RAG
Huang et al. (2014) [85]	145 RAG 72 LAG 73	Subtotal (94%) Total (6%)	90	China	24	Increased cost and operative time RAG Decreased blood loss RAG
Junfeng et al. (2014) [89]	514 RAG 120 LAG 394	Subtotal (78%) Total (22%)	45	China	22	Increased operative time, lymph node yield RAG Reduced blood loss RAG
Park et al. (2015) [90]	770 RAG 148 LAG 622	Mixed Subtotal (75%) Total (25%)	97	South Korea	24	Increased operative time RAG
Lee et al. (2015) [53]	400 RAG 133 LAG 267	Subtotal	82	South Korea	23	Increased operative time, decreased blood loss RAG Increased lymph node yield in “obese” (BMI 27) patients in RAG (RAG group was statistically younger)
Kim et al. (2016) [91]	370 RAG 185 LAG 185	Subtotal	94	South Korea (multicentre)	24	Increased operative time RAG
Okumura et al. (2016) [92]	502 RAG 370 LAG 132	Subtotal (77%) Total (23%)	85	Japan	24	Increased operative time RAG RAG is safe in the elderly (mean age 70)
Parisi et al. (2017) [18]	604 RAG 151 LAG 151 OG 302	Mixed Subtotal (74%) Total (26%)	75	Multicentre (IMIGASTRIC)	24	Increased operative time RAG Reduced LOS RAG/LAG
Yang et al. (2017) [93]	915 RAG 173 LAG 511 OG 241	Mixed Subtotal (86%) Total (14%)	88	South Korea	24	Decreased LOS MIG
Li et al. (2018) [19]	224 RAG 112 LAG 112	Mixed Subtotal (57%) Total (43%)	35	China	24	Increased operative time RAG Reduced blood loss RAG Increased cost RAG
Liu et al. (2018) [94]	235 RAG 100 LAG 135	Mixed Subtotal (58%) Total (42%)	40	China	21	Reduced LOS RAG
Gao et al. (2019) [95]	326 RAG 163 LAG 163	Mixed Subtotal (62%) Total (38%)	35	China	24	Increased operative time and cost for RAG

*RAG* robot-assisted gastrectomy, *LAG* laparoscopic-assisted gastrectomy, *OG* open gastrectomy, *LOS* length of stay, *MIG* minimally invasive gastrectomy (RAG and LAG combined)

**Table 2:** Western studies included in the meta-analyses on RAG performed to date

Pugliese et al. (2009) [68]	64 RAG 16 LAG 48	Subtotal	55	Italy	29	No difference
Caruso et al. (2011) [55]	169 RAG 29 OG 120	Subtotal (59%) Total (41%)	76	Italy	28	Increased operative time RAG Reduced blood loss and LOS RAG Reduced lymph node yield RAG
Cianchi et al. (2016) [51]	71 RAG 41 LAG 30	Distal	73	Italy	27	Increased operative time RAG Increased number of lymph nodes retrieved in RAG
Procopiuc et al. (2016) [23]	47 RAG 18 OG 29	Mixed Subtotal (44%) Total (56%)	50	Romanian	26	Increased operative time RAG
Caruso et al. (2018) [47]	39 RAG 19 LAG 20	Total	Unknown	Spain	Unknown	Increased operative time RAG Reduced blood loss and LOS RAG

*RAG* robot-assisted gastrectomy, *LAG* laparoscopic-assisted gastrectomy, *OG* open gastrectomy, *LOS* length of stay

## Long-term outcomes

### Survival

The evidence for oncological equivalence of minimally invasive gastrectomy has been published [5–7]. Because uptake of RAG is still relatively low, and the constraints imposed by the insurance systems in the Far East which mean the patient has to pay for the difference in cost compared to LAG, multicentre randomised controlled trials are not ongoing. The meta-analyses previously referenced to here, as well as the KLASS-01/02 Trials comparing LAG and OG, have not shown a significant difference in oncological outcomes between RAG, LAG or OG. The largest series on the oncological results, in terms of overall survival (OS), relapse-free survival (RFS) and recurrence patterns was published by Obama et al. [46]. This single-centre retrospective, prospectively collected, propensity-score matched study (the matching of which have been questioned some [56]) compared 313 RAG with 313 (out of 524) LAG. Total or subtotal gastrectomy was performed according to the Japanese gastric cancer treatment guidelines [57]. The median follow-up was 85 months and showed no significant difference in OS or RFS. It is important to realise, in the context of the disease stage experienced in Europe, that approximately 75% of these patients had T1N0 disease, and hence OS was > 90% for both groups.

### Functional

There are very little data on the functional outcome of RAG compared to LAG or OG. The main reason for this is that the majority of functional problems are related to the reconstruction which, where available, in the studies published to date was all performed in an identical manner to LAG using laparoscopic staplers and or extracorporeal joins. Like in laparoscopy, there has been an increasing trend towards intracorporeal anastomoses. With the recent advances in laparoscopic and robotic tri-stapling devices, this trend is expected to continue.

### Cost

The perceived additional cost associated with robotic surgery is a common argument against adopting the technology. Although the robot itself, the consumables and maintenance are a considerable financial investment, there are numerous examples, where robotic surgery has proven to be cost effective in high volume centres [58, 59] or even cost saving [60, 61], be it within certain, realistic, anticipated complication levels. Although the financial comparison between LAG and OG has not been formally assessed (although expected as part of the LOGICA trial), the cost of RAG was compared to LAG in a multicentre prospective match cohort study and found to be around $5000 higher ($13432 vs $8090) [17]. The cost assessment considered the entire expenditure of the admission; this included consumables as well as potential interventions required during the index hospital admission. Although the study was prospective, 8 out of 17 of the surgeons had relatively minimal robotic experience (fewer than 30 cases, including one surgeon who had only performed 4 RAG) and the learning curve effect can, therefore, not be excluded and should be considered whenever conclusions are drawn from, what is to date, the only multicentre prospective comparative study between RAG and LAG. In one of the largest single-centre case series to date (243 cases), Woo et al. [62] discuss the complexity surrounding the analysis of cost when comparing RAG and LAG. Because most the case series come from the Far East, the role of healthcare insurance plays an important part in the decision of a patient to have RAG or LAG; the patient are commonly expected to pay the difference between RAG and LAG as the insurance will not cover this. This, of course, means any cohort comparisons are non-randomised and potentially biased. For example, the matched cohorts are statistically different in age (RAG cohort is younger) and reconstruction [RAG majority Billroth II (51%) vs LAG Billroth I (54%)]. Beyond this, it has historically been challenging to truly calculate potential long-term cost savings for a given procedure. In colorectal surgery for example, the potential reduction in stoma rates for robotic low rectal resections could have a huge consequence for the health economics associated with the procedure, although this benefit is currently not proven [63]. Equally, reduced length of ICU admission or overall stay has cost effects [64]. Uyama et al. showed in their prospective study that complications are associated with increased cost [45]. The same study showed a significant reduction in complications (compared to a historic LAG group) and subsequently improved quality of life. These findings combined were sufficient to the Ministry of Health, Labour, and Welfare in Japan to recognise RAG as a valid minimally invasive technique from a health insurance perspective.

In the context of cancer surgery, especially gastric surgery where neoadjuvant therapy is the standard of care for most tumours, costs need to be contextualised; the cost of 4 courses of FLOT is in the order of $2000 [65]. However, drugs like Trastuzamab given in the context of Her2 + ve advanced gastric cancer attract far greater costs ($70000 annually) for a mean survival benefit of 2.7 months [66], vastly eclipsing the increased costs currently associated with robotic surgery. Nonetheless, in the absence of alternative high level evidence for the benefits of robotic surgery in certain procedures, the costs of robotic consumable and hardware are currently a major argument against mainstream implementation in many healthcare systems. The development of new robots has already seen a change in both design (open consoled, modular systems), but also the business models used to alter the associated costs with robotic surgery [67]. For example, Cambridge Medical Robotics from the UK have adopted alternative costing models which cover maintenance, instruments and even assistants as a comprehensive package, beyond solely charging for hardware.

### RAG in Europe

To date only five European RAG cohort studies have been published [23, 47, 51, 55, 68]. The total number of RAG cases included in these is 123. Unit volume should, therefore, be considered carefully when interpreting the available data. The mean BMI of the combined European cohorts is 28 kg/m^2^, which according to WHO guidelines is overweight (the term obese is frequently incorrectly used in the RAG literature to include anyone with a BMI greater than 25). This compares to a mean BMI of 23 kg/m^2^ in the Asian cohorts. The stage of disease at the time of surgery is more advanced in the European cohort > T2N1 in 55–75%; only one Asian study reports a larger proportion of advanced tumours [19]. Gastroesophageal junctional adenocarcinomas are particularly relevant in this context; these tumours are more prevalent in Western countries [69] and typically present with more advanced disease and lymph node involvement [70]. Reports of increased prevalence in Far Eastern countries have become more commonplace [71], but the percentage of total gastrectomies in the Western studies is higher which, although not specifically commented on, would imply a greater proportion of proximal/junctional tumours. Interestingly, more recent reports from the Far East have started to include larger numbers of total gastrectomies—whether this reflects a shift in disease patterns or a greater willingness/aptitude at the procedure to therefore operate on more proximal tumours, again, is not specifically commented on. The 8th edition of the American Joint Commission on Cancer (AJCC) staging manual [72] redefined GEJ cancers. In this, more recent type III are now considered gastric tumours. Consequently, many centres now treat these tumours accordingly in a neo-adjuvant setting with FLOT [73]. Interestingly, the surgical management, however (total/extended gastrectomy versus esophagectomy), remains controversial and is the subject of a proposed prospective multicentre trial; the CARDIA trial [74].

The relatively limited European RAG comparative cohort studies that have been reported to date indeed show differences in the patient characteristics as illustrated above. Nonetheless, the outcomes in these studies in terms of overall outcomes, complications and technical aspects such as operative time and blood loss, despite being relatively small cohorts, do reflect those observed in the Far East.

## The future

The available evidence to date shows RAG is a safe and oncologically sound alternative to LAG or OG. The benefits of RAG, thus far, have been relatively minor and appear to come with an increased cost and longer operation time based on available cohort studies. The majority of the studies include distal gastrectomies for early tumours performed by surgeons relatively early on in their learning curve. As such, some caution must be exercised when translating the current evidence to a European population. Ojima et al. have started a phase III single-centre randomised controlled trial comparing LAG vs RAG for stage I–III gastric cancer [75], which has been recruiting since April 2018.

Benefits of robotic systems not discussed here, or formally studied in the context of RAG, include the use of ICG to assess vascularity [76] and (sentinel) lymph nodes [77]. Inclusion of artificial intelligence and machine learning to aid the surgeon in these complex procedures are coming on the horizon [78]. A recent phase I/II trail [79] has shown reduced port robotic distal gastrectomy to be safe and feasible.

There has also been a global push to prospectively collect clinical information of patients undergoing RAG by means of the IMIGASTRIC project [80], which has been mirrored by other oesophagogastric operations such esophagectomy in the context of UGIRA [81]. These database initiatives are key to monitor and share experiences of a seismic change in surgical practice that is here to stay.
